# Vehicle Detection in Aerial Images Based on Region Convolutional Neural Networks and Hard Negative Example Mining

**DOI:** 10.3390/s17020336

**Published:** 2017-02-10

**Authors:** Tianyu Tang, Shilin Zhou, Zhipeng Deng, Huanxin Zou, Lin Lei

**Affiliations:** College of Electronic Science and Engineering, National University of Defense Technology, Changsha 410073, China; ttywhu@163.com (T.T.); zpdeng@whu.edu.cn (Z.D.); hxzou2008@163.com (H.Z.); alaleilin@163.com (L.L.)

**Keywords:** vehicle detection, hyper region proposal network, convolutional neural networks, hard negative example mining

## Abstract

Detecting vehicles in aerial imagery plays an important role in a wide range of applications. The current vehicle detection methods are mostly based on sliding-window search and handcrafted or shallow-learning-based features, having limited description capability and heavy computational costs. Recently, due to the powerful feature representations, region convolutional neural networks (CNN) based detection methods have achieved state-of-the-art performance in computer vision, especially Faster R-CNN. However, directly using it for vehicle detection in aerial images has many limitations: (1) region proposal network (RPN) in Faster R-CNN has poor performance for accurately locating small-sized vehicles, due to the relatively coarse feature maps; and (2) the classifier after RPN cannot distinguish vehicles and complex backgrounds well. In this study, an improved detection method based on Faster R-CNN is proposed in order to accomplish the two challenges mentioned above. Firstly, to improve the recall, we employ a hyper region proposal network (HRPN) to extract vehicle-like targets with a combination of hierarchical feature maps. Then, we replace the classifier after RPN by a cascade of boosted classifiers to verify the candidate regions, aiming at reducing false detection by negative example mining. We evaluate our method on the Munich vehicle dataset and the collected vehicle dataset, with improvements in accuracy and robustness compared to existing methods.

## 1. Introduction

**Figure 1:**
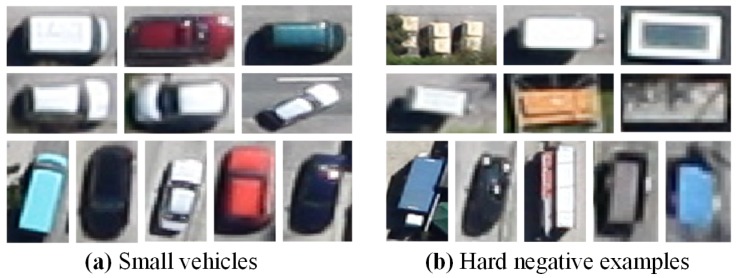
Two challenges for vehicle detection in aerial images. (**a**) small vehicles; (**b**) hard negative examples.

In very high resolution (VHR) remote sensing images, vehicle detection is an indispensable technology in both civilian and military surveillance, e.g., traffic management, urban planning, etc. Therefore, vehicle detection from aerial images has attracted significant attention worldwide [1,2,3,4,5]. However, automatic vehicle detection in aerial images still has a lot of challenges due to the relatively small size and variable orientation of vehicles (Figure 1a). In addition, real-time detection in such large-scale aerial images with intricate backgrounds (Figure 1b) also increases the difficulties.

In previous studies, the existing vehicle detection methods in aerial images are mostly based on sliding window search and manual features or shallow-learning-based features [6,7,8,9,10,11]. The work of [2] is worth mentioning here, as the authors presented an approach that could detect vehicles with type and orientation attributes on large-scale aerial images without any geo-reference information available. This method employed a fast binary detector using integral channel features (ICFs) and an AdaBoost classifier in a soft-cascade structure to detect the location of the vehicles. Then, a histogram of oriented gradient (HOG) features was used to further classify the orientations and type of the vehicle, resulting in both rapid and effective detection performance. However, this method has some drawbacks. Firstly, hand-crafted features or shallow-learning based features influence the representational power, as well as the effectiveness of vehicle detection. Secondly, the sliding window technique leads to heavy computational costs.

In the field of computer vision, region convolutional neural network (R-CNN) based detection methods have achieved great success in nature scene images [12], especially Faster R-CNN [13]. Faster R-CNN employs a fully convolutional region proposal network (RPN) to generate object-like regions, and a classifier after RPN to further infer the candidate regions. Considering the powerful feature representation and fast speed, Faster R-CNN performs much better than the traditional sliding window based methods. However, directly utilizing it for vehicle detection in such large aerial imagery has many challenges, due to the big differences between aerial images and nature scene images. The differences and challenges are as follows: (1) vehicles (only 30×12 pixels) in aerial images (with the size of 5616×3744 pixels) are relatively smaller than those in nature scene images, thus increasing the difficulty of localization; (2) due to the large size and wide view of the aerial images, their backgrounds are more intricate than the nature scene images. Accurate vehicle detection in such aerial images is hard; (3) the aerial images are much larger than the nature scene images (approximately with the size of 700×700 pixels), increasing difficulties for rapid detection. In addition, the training data for vehicle detection in aerial images is much less, causing an over-fitting problem for region CNN-based methods. Considering all the challenges mentioned above, we argue that two reasons lead to Faster R-CNN’s poor performance. Firstly, region proposal network (RPN) in Faster R-CNN is not good enough to accurately detect small-sized vehicles, due to the relatively coarse feature maps. Secondly, the classifier after RPN cannot distinguish vehicles and complex backgrounds well, due to the lack of hard negative example (similar to vehicles, see Figure 1) mining process.

**Figure 2:**
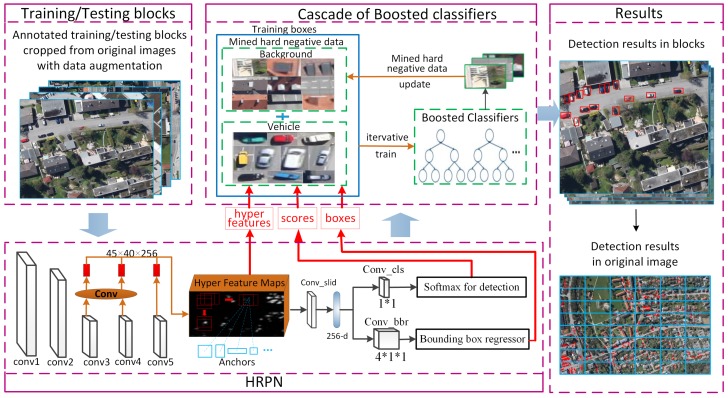
Proposed vehicle detection framework. Original large scale images are cropped into small scale blocks for training and testing.

To address these problems, in this paper, we propose an accurate and robustness vehicle detection framework (see Figure 2). Our method contains two parts: a hyper region proposal network (HRPN), aiming at predicting all of the possible bounding-boxes of vehicle-like objects with high recall rate, and a cascade of boosted classifiers to further verify the detection results from HRPN with high precision. Specifically, our HRPN is based on RPN. However, HRPN combines rich-detail feature maps of shallow layers with coarse-detail feature maps of deep layers, which is more suitable for small object detection than RPN. In addition, we replace the classifier after RPN by a cascade of boosted classifiers, which can reduce false detection by mining hard negative examples.

Moreover, limited annotated training data causes an over-fitting problem, and large-scale aerial images increase the difficulty of rapid detection. To overcome the two problems above, we crop the original large-scale training aerial images into image blocks, and augment the number of them by rotation. In addition, the testing aerial images are also cropped into blocks. The vehicles’ locations can be acquired when the blocks with testing results are stitched back together. Compared with [2], our method is more accurate and more robust without setting the size of the detection window in advance. Compared with Faster R-CNN [13], our method has better location accuracy and less false detection. Moreover, we successfully test our method on unmanned aerial vehicle (UAV) images and pansharpened color infrared (CIR) images, proving the robustness of our method. The main contributions of our work is: we combine a cascade of boosted classifiers with HRPN, which improves the classification accuracy by hard negative example mining.

This paper is organized as follows: Section 2 discusses related works. The proposed method is detailed in Section 3. Section 4 reports the experimental results. Finally, Section 5 concludes the paper.

## 2. Related Work

According to the existed object detection methods, the detection task can be divided into three main parts: generation of candidate regions, feature extraction and classification.

For generation of candidate regions, most of the existing vehicle detection methods employ a sliding-window search algorithm [2,3,7,8]. These methods need to set different sizes of windows to traverse the entire image, or use a fixed-size window to traverse the zoom images. Therefore, these sliding-window search based methods have a high complexity of time and generate a large number of redundant windows. Compared with the sliding-window based methods, region-proposal based methods reduce the computational costs. Traditional methods generate region proposals by merging segments that are likely be included in objects, e.g., super-pixels [14], saliency [15], selective search [16], etc. Nevertheless, the human-designed generator for region proposals is still time-consuming. Recently, CNN based methods have been widely used in region proposals, such as Deepbox [17] and RPN [13]. These methods can generate candidate regions with deep learning features, resulting in promising performance and speed.

For feature extraction, hand-crafted features are widely used in vehicle detection. Shao et al. [7] used Haar-like features and local binary patterns (LBP) for vehicle detection. Moranduzzo et al. [3] made use of scale-invariant feature transform descriptors (SIFTs). Kluckner et al. [8] and Tuermer et al. [18] adopted a histogram of oriented gradient (HOG) features, while Liu [2] employed a fast binary detector using ICFs. However, these hand-crafted features are not good enough at separating cars from the background in complex environments. Recently, region CNN-based detection methods have achieved great success in nature scene images, owing to their powerful feature representation. The most popular is region-based convolutional neural networks (R-CNN) [19] and their improved methods Fast R-CNN [20] and Faster R-CNN [13]. All of them achieve state-of-the-art performance.

For classification, Shao et al. [7] and Moranduzzo et al. [3] used hand-crafted features with a support vector machine (SVM) for candidate region classification. However, AdaBoost gradually replaced SVM due to its good performance. Kluckner et al. [8], Tuermer et al. [18] and Liu et al. [2] employed an AdaBoost classifier to verify candidate regions. This research demonstrates that the bootstrapping strategy, which mines hard negative examples and reweights examples for iterative training, improves the classifier considerably by reducing the number of false classification.

Considering all three parts of the detection, the region CNN-based detection methods have the best performance. R-CNN uses a selective search algorithm to generate object-like regions, and then extracts deep features for classification by SVM. In order to increase the speed and accuracy of detection further, Fast R-CNN employs a multi-task network for classification and bounding box regression, combining feature extraction and classification into one process. Nevertheless, their human-designed region proposal generator is still time-consuming. Thus, Faster R-CNN employs RPN to generate object-like regions and a CNN-based classifier to further infer the candidate regions. Faster R-CNN is an end-to-end method, achieving near real-time detection with state-of-the-art performance. However, it still has some drawbacks when applied to vehicle detection in aerial images. RPN is not suitable for small objects, owing to the coarse feature maps it uses. Meanwhile, the classifier is not good enough to distinguish objects from complex backgrounds, due to the lack of hard negative examples mining process.

We take the advantages of Faster R-CNN and hard negative example mining to propose our method, achieving state-of-the-art performance.

## 3. Proposed Method

The framework of our vehicle detection method is illustrated in Figure 2. For training, we crop original large-scale images into blocks and augment the number of image blocks by rotation with four angles (i.e., $0^{\circ }$, $90^{\circ }$, $180^{\circ }$, and $270^{\circ }$). Then, HRPN takes all the training image blocks as input for training and produces candidate region boxes, scores and corresponding hyper features. Finally, the outputs of HRPN are used to train a cascade of boosted classifiers, and the final classifier is obtained.

For testing, a large-scale testing image is cropped into image blocks. Then, HRPN takes these image blocks as input and generates candidate region boxes as well as hyper feature maps. The final classifier verifies these boxes using hyper features. Finally, all the detection results of blocks are stitched together to recombine the original image.

### 3.1. Hyper Region Proposal Network

**Figure 3:**
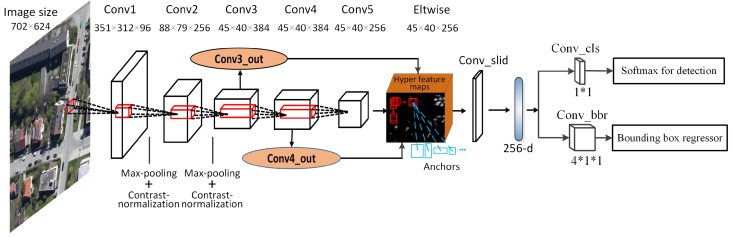
Architecture of Hyper Region Proposal Network (HRPN). For 702×624 images, the feature maps size of Conv1-Conv5 are 351×312×96, 88×79×256, 45×40×384, 45×40×384, 45×40×256, respectively.

There are two kinds of RPN structures: one is based on the Zeiler and Fergus model (ZF) [21], and the other is based on the VGG model [22]. Owing to the limited training data and video memory, we choose the relatively few parameter ZF-based RPN as the building block of our HRPN. Different from RPN, we combine the output feature maps of the last three convolutional layers to achieve a concatenated feature map. The reasons for our improvement are as follows: Ghodrati [23] indicates that the deeper convolutional layers can get high recall but poor localization in detection, and the lower convolutional layers can get more accurate localization but low recall. In addition, the Fully Convolution Network (FCN) achieves great performance in segmentation [24], in which the authors combine a high layer with a low layer for segmentation. Therefore, these imply that the combination of shallow features and deep features will result in better detection results. Figure 3 shows the architecture of HRPN.

#### 3.1.1. Architecture

HRPN is a full convolutional network to generate candidate regions. The first convolutional layer (Conv1) takes the training images as input and has 96 kernels of size 7×7×34 with a stride of two pixels. The second convolutional layer (Conv2) takes the output of the previous convolutional layer as input and filters it with a stride of two pixels by 256 kernels of size 5×5×96. The contrast normalization and max pooling layers are only configured after the first two convolutional layers. The third (Conv3), fourth (Conv4), and fifth (Conv5) convolutional layers are directly connected to each other, having 384 kernels of size 3×3×384, 384 kernels of size 3×3×384, and 256 kernels of size 3×3×256, respectively. We compute hyper feature maps from three convolutional layers (namely Conv3, Conv4 and Conv5), which have the same size but different levels of detail information. In addition, two convolutional layers (Conv3_out and Conv4_out) with kernel size of 1×1×256 are added on the top of Conv3 and Conv4 feature maps, respectively, to compress them into the same number as conv5. Finally, we synthesize these three feature maps and get one single output cube, which we call hyper feature maps. The “Eltwise” layer is used to complete the operation, which simply adds the three feature maps together.

In order to generate candidate regions, we use the sliding window operation on hyper feature maps. This operation is implemented by a 3×3 convolutional layer, namely “Conv_slid”. The parameters of weight are initialized by “gaussian”, and the parameters of bias are initialized by “constant”. At each sliding window location, we simultaneously predict multiple region proposals associated with different scales and aspect ratios (namely anchors, see blue boxes in Figure 3). As the size of vehicle is approximately 35×35, we adopt anchors of three scales of $30^{2}$, $40^{2}$, and $50^{2}$ pixels, and three aspect ratios of 1:1, 1:2, and 2:1. For 256 feature maps in total, we can extract a 256-d feature vector for each region proposal. Afterwards, these region proposals and their corresponding features are fed into two sibling 1×1 convolutional layers for box-classification and box-regression, respectively (namely conv_cls and conv_bbr). The first sibling layer outputs a vehicle-like score $p^{c}$, and the second sibling layer outputs the coordinates vector loc=(x,y,w,h) of each predicted region after bounding box regression. *x* and *y* represent the top-left coordinates of the predicted region, whereas, *w* and *h* denote the width and height of the predicted region.

#### 3.1.2. Training HRPN

To facilitate training by limited size of the training set, we use a pre-trained ZF model [13,25] based on ImageNet [26] to initialize the parameters of five convolutional layers, and then domain-specifically fine-tuned with a smaller learning rate. Throughout the training process for HRPN, we have 70k iterations. During each iteration, we process a batch of the labeled image blocks into the network, and obtain region proposals for each image block. For each batch, the number of image blocks and predicted regions are $N_{cls}$ and $N_{bbr}$, respectively. If a predicted region has the Intersection-over-Union (IoU) bigger than 0.7 with the ground truth box, we assign a positive label to it (${p_{i}}^{*}=1$). However, if the IoU ratio of a predicted regions is lower than 0.1 for all ground-truth boxes, we assign a negative label to it (${p_{i}}^{*}=0$). Then, the remaining regions are discarded. The IoU ratio is defined as follows:

(1) $$a=\frac{\mathrm{area}(B_{p}\cap B_{g})}{\mathrm{area}(B_{p}\cup B_{g})},$$

where $area(B_{p}\cap B_{g})$ represents the intersection of the vehicle proposal box and ground truth box, and $area(B_{p}\cup B_{g})$ denotes their union.

All of the positive and negative region proposals and their corresponding labels are fed into the loss function. In addition, a multi-task loss function is used to update the parameters of the network, aiming at minimizing the error of classification and localization. We use $L_{cls}$ as the softmax loss fuction for the classification of vehicles and backgrounds, and $L_{bbr}$ as the box-regression loss. Just like [13], the loss function is defined as

Equation (2):

(2) $$\begin{array}{c} L_{HRPN}\left(p_{i},loc_{i}\right)=\frac{1}{N_{cls}}\sum_{i}L_{cls}\left(p_{i},p_{i}^{*}\right)+\lambda \frac{1}{N_{bbr}}\sum_{i}p_{i}^{*}L_{bbr}\left(loc_{i},lo{c_{i}}^{*}\right), \end{array}$$

where *i* is the index of a batch. $p_{i}$ is the vehicle-like score for each predicted region, and ${p_{i}}^{*}$ is the ground-truth label. *λ* is the balance parameter. During each iteration, the number of positive and negative region proposals are the same. Therefore, we set λ=2 to weight both $L_{cls}$ and $L_{bbr}$ terms equally. $L_{bbr}$ denotes a smooth $L_{1}$ loss [13], which is the same as those in Faster R-CNN. It is defined as Equation (3):

(3) $$\begin{array}{c} L_{bbr}\left(loc_{i},loc_{i}^{*}\right)=f_{L1}\left(loc_{i}-loc_{i}^{*}\right),\\ f_{L1}\left(x\right)=\{\begin{array}{c} 0.5x^{2},if\left|x\right|<1,\\ |x|-0.5,otherwise, \end{array} \end{array}$$

where $loc_{i}$ is the coordinates vector of the predicted region, and $lo{c_{i}}^{*}$ is the coordinates vector of target ground-truth bounding-box. When the training of the HRPN is finished, we generate approximately 300 highly overlapped candidate region boxes of each test block. To reduce redundancy, non-maximum suppression (NMS) is adopted in the proposed regions based on the vehicle confidence score $p^{c}$. Finally, the remaining vehicle-like regions and their scores are used as the initial data for the boosted classifiers that follow.

### 3.2. Cascade of Boosted Classifiers

**Figure 4:**
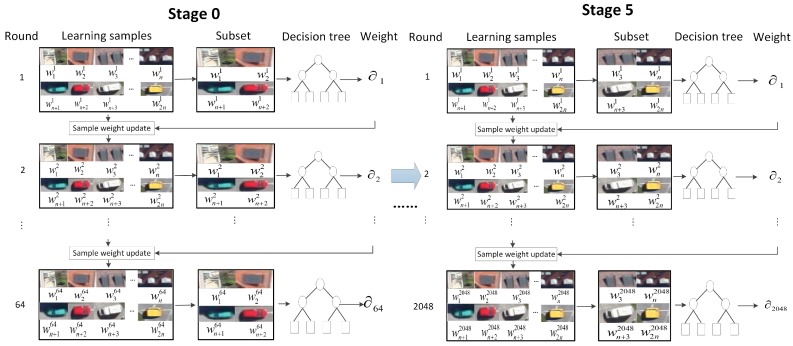
Training process of the cascade of boosted classifiers. We adopt the cascade of six stages, and employ {64, 128, 256, 512, 1024, 2048} weak classifiers in each stage.

The HRPN has proposed candidate regions, scores and corresponding hyper features, which are used as training data for cascade of boosted classifiers. In this section, a candidate region is considered as a positive sample if it has an Intersection-over-Union (IoU) ratio bigger than 0.8 with the ground truth box, and the negative lower than 0.3. Initially, all the positive samples and the same number of randomly chosen negative samples from candidate regions are used as the training data. The training process of the cascade of boosted classifiers is illustrated in Figure 4.

We set six stages for training. For stage 0, we use 10k positive examples and 10k negative examples to train the 64 weak classifiers. Then, all the training data of stage 0 plus 1k new negative examples are used to train the classifiers of stage 1. Each stage uses the reweighted examples from previous stage and the newly added negative examples for training.

Moreover, we use {64, 128, 256, 512, 1024, 2048} weak classifiers in each stage. A shallow decision tree is taken as a weak classifier. Then, the RealBoost algorithm [27] is used to constitute strong classifiers from weak classifiers—namely, boosted classifiers. In stage 0, we set the weak classifier $f_{0}$ following the form of RealBoost:

(4) $$\begin{array}{c} f_{0}=\frac{1}{2}\log\frac{s_{i}}{1-s_{i}} \end{array},t_{i}=1,2\cdots T_{i},$$

where $s_{i}$ is the score of candidate region from HRPN. Following [27], the weak classifier in each round of other stage is defined as Equation (5):

(5) $$\begin{array}{cc} f_{t_{i}}=\frac{1}{2}\log\frac{1-e_{t_{i}}}{e_{t_{i}}} & i=1,2\cdots 5 \end{array},t_{i}=1,2\cdots T_{i},$$

where, in stage *i* round $t_{i}$, $e_{t_{i}}$ is the error rate of the decision tree, and $T_{i}$ is the number of tree. In each round, we choose a subset of the training samples to train the classifier and the samples are reweighted according to the classification results. Specifically, the weights of the correctly categorized samples are reduced and the weights of the incorrectly categorized samples are increased. Thus, hard negative examples are mined and added into the training set.

After $T_{i}$ times iterative training, the weights of training data are updated and $T_{i}$ weak classifiers are obtained. The final detector $F_{i}$ of stage *i* is defined in Equation (6):

(6) $$\begin{array}{cc} F_{i}=\frac{1}{T_{i}}\sum_{t=1}^{T_{i}}\partial_{t_{i}}f_{t_{i}} & i=1,2\cdots 5,t_{i}=1,2\cdots T_{i}, \end{array}$$

where $\partial_{t_{i}}$ is the weight of the decision tree.

After being trained by all stages, a classifier consisting of 2048 weak classifiers is obtained and used to classify candidate regions. Our implementation is based on [28].

### 3.3. Implementation Details

In this paper, the structures and layers of HRPN are based on the existing ZF model, and the kernel sizes are the same as the ZF model. In HRPN, an input image will be resized to n×m pixels before training and testing (1053×936 for image blocks with the size of 702×624 pixels in the Munich Vehicle data set). Therefore, in order to achieve better results, we need to set different n×m for a new dataset with different sizes of images. In addition, each batch consists of one image and 120 randomly selected anchors for training.

To determine the parameters of the anchor, a comparison experiment is done. We evaluate the mean average precision (mAP) under different scales and aspect ratios of anchors. The results are shown in Table 1. As can be seen from the table, the results of one scale and three different ratios are better than that with one scale and one ratio. The results of three scales and three ratios are similar to those with one scale and three ratios. Taking all the results into account, we select the parameter of anchors with three scales and three ratios.

Other parameters of HRPN are the same as in Faster R-CNN [13].

## 4. Experimental Results

In this section, we analyze our vehicle detection method. Experiments were implemented based on the deep learning framework Caffe [29] and run on a PC with Intel core i7-4790 CPU, a NVIDIA GTX-960 GPU (NVIDIA, Santa Clara, CA, USA), (2 GB video memory), and 8 GB of memory. The operating system was Ubuntu 14.04 (Canonical, London, UK).

### 4.1. Data Set

#### 4.1.1. Data Set Description

Two data sets were used in these experiments. The Munich Vehicle data set [30] was collected over the city Munich, Germany. It contains 20 aerial images captured by a DLR 3K camera system [1] at a height of 1000 m above ground. The collected vehicle data set contains a total of 17 UAV images and 85 very-high-spatial-resolution pansharpened color infrared (CIR) images. The first eight UAV images [3,4] were taken over the Faculty of Science of the University of Trento (Italy) and the other nine UAV images were captured over the city Changsha, China. Seventeen UAV images have a spatial resolution of approximately 2 cm, and 85 CIR images were acquired from an Northwestern Polytechnical University (NWPU) VHR-10 data set [31,32], which are downloaded from the Vaihingen data set [33] with a spatial resolution of approximately 0.08 m.

#### 4.1.2. Data Preparation

**Figure 5:**
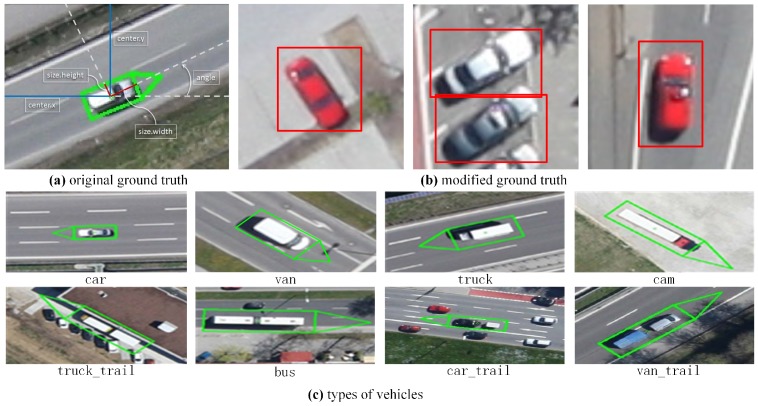
Ground truth of the original Munich dataset, our modified ground truth and types of vehicles.

The Munich Vehicle data set is annotated with rotated bounding boxes for eight types of vehicles (see Figure 5a,c). Following [2], the first ten images are used for training and the other for testing. In the training dataset, the number of each type of vehicle is shown in Table 2.

Following [2], we use two vehicle classes, the composition of “ca” and “van” as car and the “truck” and “cam” as truck. There are 3418 cars and 65 trucks annotated in the training images, and a few other types of vehicles (car_trail, van_trail, truck_trailer and bus). In the test set, there are 5799 cars and 93 trucks. To make the distribution of training data reasonable, we select cars and trucks to train our model. Due to the limited size of the training set and video memory, each original aerial image (5616×3744 pixels) is cropped into 11×10 image blocks (702×624 pixels) with overlap. The blocks without vehicles are discarded and the remaining image blocks are rotated with four angles (i.e., $0^{\circ }$, $90^{\circ }$, $180^{\circ }$, and $270^{\circ }$). Additionally, as the number of trucks is much less than cars, we copy the image blocks that contain trucks 30 times. Meanwhile, we create corresponding annotation files for each block, which contain coordinates vectors of bounding box and type. Thus, based on the ten training images, we reconstructed a new training set with 2120 images and corresponding annotation files.

Specifically, the annotation information of each image block is loaded from the original dataset, according to its location in the original image. Then, we update the coordinate of each bounding box, based on the relationship between image blocks and the original image. Moreover, the original bounding boxes are rotated, but our method can only generate candidate boxes with no angles. Therefore, we compute bounding boxes without rotation, according to the center point’s coordinates, height and width of the bounding boxes in original annotations ($center_{\cdot }x$, $center_{\cdot }y$, $size_{\cdot }width$, $size_{\cdot }height$, see Figure 5a). For example, when the angle between $65^{\circ }$ and $115^{\circ }$ or $-65^{\circ }$ and $-115^{\circ }$, the upper left corner’s coordinates of bounding boxes are $center_{\cdot }x$ minus half length, and $center_{\cdot }y$ minus half width. The width and height of the bounding box are unchanged (see Figure 5b).

For large-scale test images of the Munich data set, we crop each image into 48 blocks. To avoid missing detection of vehicles on the cross image block boundaries, we set the overlap of the adjacent image blocks as 50 pixels. The 48 image blocks are detected separately and then stitched together to recombine the original image.

The collected vehicle data set contains 85 CIR images with annotations. Therefore, we manually label the 17 UAV images with bounding box and type. This dataset is just used for testing the robustness of our method.

### 4.2. Results for Munich Images

We adopt four widely used measures to quantitatively evaluate the performance of our method in the following—namely, recall rate, precision-recall curve (PRC), mean average precision (mAP), and F1-score. The recall rate measures the fraction of correctly identified positive detections and true positive detections, while the precision measures the fraction of correctly identified positive detections and predicted positive detections. The mAP metric is measured by the area under the PRC. The higher the value of mAP, the better the performance. Moreover, F1-score is defined as:

(7) $$\begin{array}{c} F1-\mathrm{score}=\frac{2\times \mathrm{recall}\times \mathrm{precision}}{\mathrm{recall}+\mathrm{precision}} \end{array}$$

#### 4.2.1. The Importance of Hyper Features

**Figure 6:**
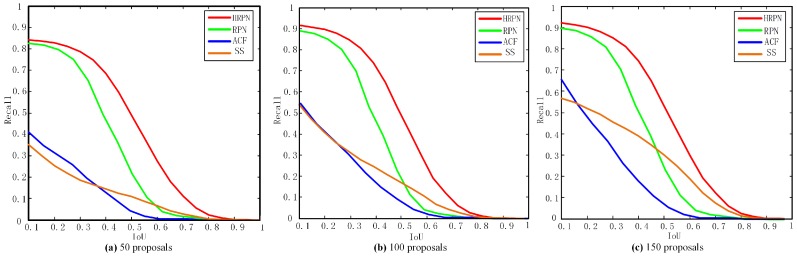
Recall versus Intersection-over-Union (IoU) threshold on the Munich Vehicle test set. (**a** 50 region proposals; (**b**) 100 region proposals; (**c**) 150 region proposals.

Firstly, we evaluate the good performance of HRPN by recall rate. We compare HRPN with the state-of-the-art method RPN [13], ACF detector (aggregated channel features, ACF) [2], and selective search [16] in terms of proposal quality. The ACF detector is considered as the baseline method. The curves of recall rates for the four methods under different IoU thresholds are plotted. In Figure 6, we evaluate 50, 100, and 150 region proposals per image block in order to see the recall rates changes with the IoU. The N proposals per image indicates the top-ranked N proposals based on the scores. The plots show that our method really improves the recall rate compared to RPN, which only uses the last convolutional layer to compute feature maps. Specifically, with 100 region proposals, HRPN gets 85.7% recall, outperforming RPN by 13.29 points, and is much better than ACF, Selective Search (SS) with IoU 0.3. With 50 proposals and 150 proposals, our HRPN also exceeds RPN, ACF and SS by a large margin with different IoUs. Moreover, the CNN-based methods (HRPN and RPN) get much higher recall than those without CNN. With IoU thresholds increasing, the recall curve of RPN drops more sharply than our HRPN with the same region proposals. With the number of region proposals increasing, recall rate for the corresponding method increases with the same IoU. Overall, HRPN can generate candidate regions with high recall, which is desirable in object detection.

Moreover, we choose a loose IoU [13] (0.3 for the final detection) for such small vehicles. If a predicted candidate region has an IoU bigger than 0.3 with the ground truth box, it is considered to be the correct detection result.

**Figure 7:**
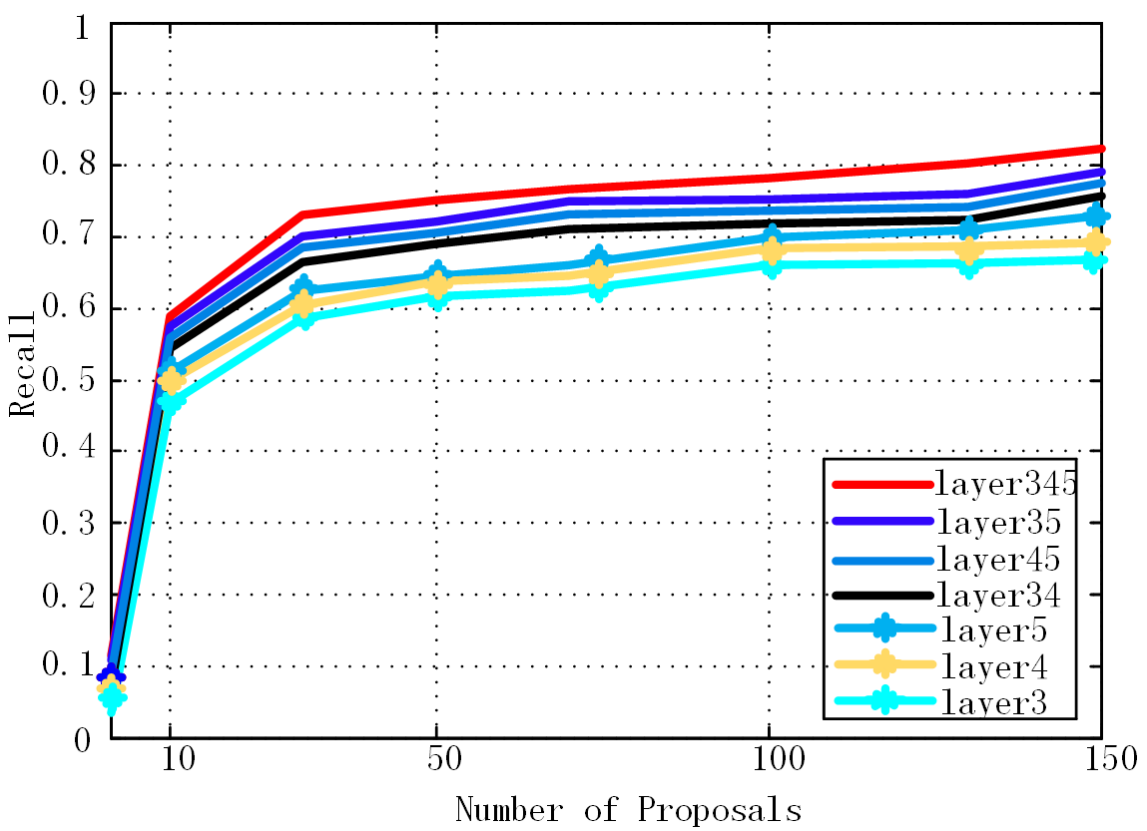
Recall versus number of proposals for different combining layers (IoU = 0.3).

Secondly, we investigate which layers should be combined. In Figure 7, we evaluate recall rate versus a different number of proposals for different combining layers. In Table 3, we evaluate the detection performance with different combining layers (region proposal number is 100 and IoU is 0.3). In addition, the recall rate and mAP in both Figure 6 and Table 3 are calculated when a boost of classifiers are added. We train all the networks with the same configuration as RPN [13] and only extract features from different combining layers for a cascade of boosted classifiers. The results in Figure 6 and Table 3 show that the combination of layers 3, 4 and 5 has the best performance. The combination of two or three layers has better results than a single layer in both recall and AP. In addition, the last layer has better performance than the other shallow single layers in detection.

#### 4.2.2. Analysis for a Cascade of Boosted Classifiers

**Figure 8:**
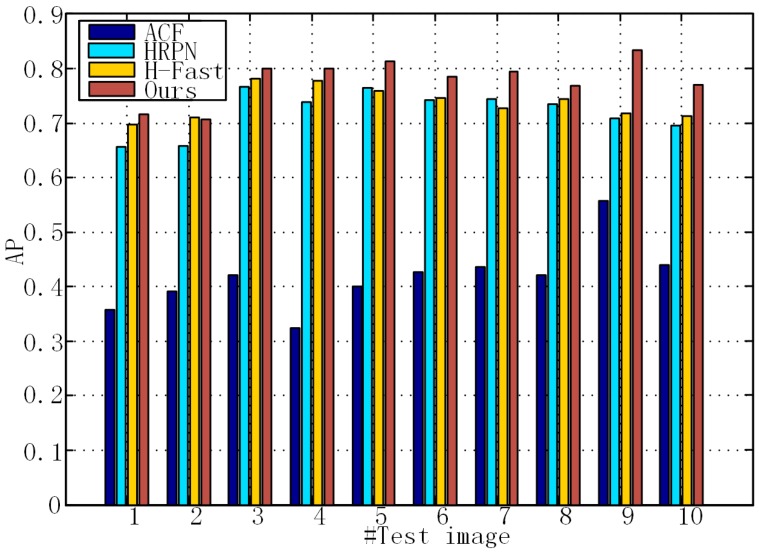
Performance comparisons of the proposed method, HRPN, H-Faster (HRPN + Fast R-CNN) and aggregated channel features (ACF) detector in terms of mAP values in 10 test images.

To verify the importance of mining hard negative samples, we compare the detection AP of HRPN alone, HRPN + Fast R-CNN (H-Fast) and HRPN + Cascade of boosted classifiers (Ours), as shown in Figure 8. According to [2], the results of ACF are considered the baseline. The histogram shows that our method has a higher AP than HRPN and H-Fast. Specifically, in image 9, our method gets an AP of 83.9%, outperforming HRPN by 12.96 points and H-Fast by 11.48 points. These results prove that the hard example mining process leads to a better identification ability. Furthermore, these results also indicate that CNN-based methods have much more impressive performance than the state-of-the-art traditional methods.

#### 4.2.3. Comparisons

We compare our method with state-of-the-art detection methods. Table 4 shows the numerical comparison results of four methods (ACF, Faster R-CNN, H-Fast and Ours) for the Munich data set. The ACF detector is the method that [2] uses. The best performances are highlighted in bold. It can be observed that our proposed method achieves the best performance in terms of recall rate and F1-score. Faster R-CNN achieves a much better performance than ACF. Compared with Faster R-CNN and H-Fast, our proposed method based on a hyper feature map can improve the recall rate effectively. Compared with H-Faster, our method has less false positives and higher F1-scores due to the hard example mining and iterative training process.

The average time spent on vehicle detection is 81 ms for each image block, respectively. Specifically, it spends 63.9 ms on HRPN, including a proposal of candidate regions and NMS. Meanwhile, the time for the cascade of classifiers is 17.9 ms.The comparisons show that the speed of our method is comparable. Furthermore, using advanced GPU with higher video memory, we can crop large aerial images into a lower number of image blocks and make the detection process parallel, which will accelerate the speed of detection.

**Figure 9:**
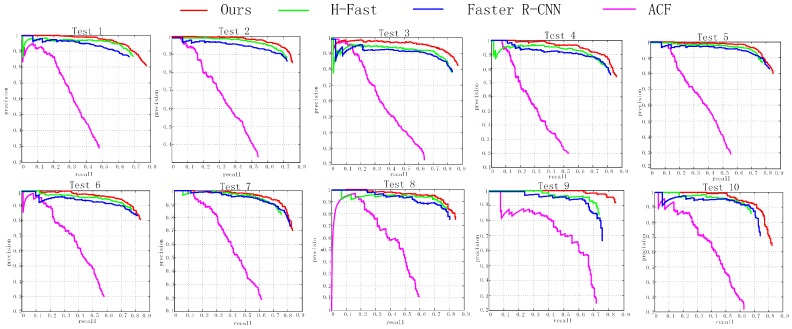
precision-recall curve (PRC) of the proposed method and other state-of-the-art approaches for vehicle detection in 10 test images, respectively.

Quantitative comparison results of ten test large scale images measured by PRCs are shown in Figure 9. We can observe that the performances of CNN-based detection methods are significantly improved over the ACF detector. The superior performance of the CNN-based methods demonstrate the high superiority of the learning based region proposal network compared with the human-designed region proposal algorithms for extracting good vehicle-like regions, which is a critical task for vehicle detection.

**Figure 10:**
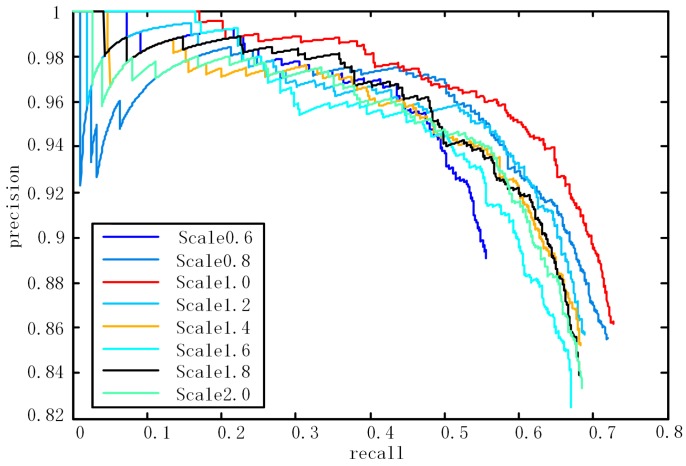
Performance after rescaling the image with different factors.

In addition, to further validate the ability of our method for vehicle detection in aerial images with different scales, we resized the image for the test but not the training. These detection results of scaled test image 2 are shown in Figure 10. Our detector performs best on the same scale as it was trained. Regardless of whether the resolution is increased or decreased, the performance remains comparable. However, if the resolution is increased or decreased under a larger scale factor, our method does not perform well.

### 4.3. Results of the Collected Vehicle Images

**Figure 11:**
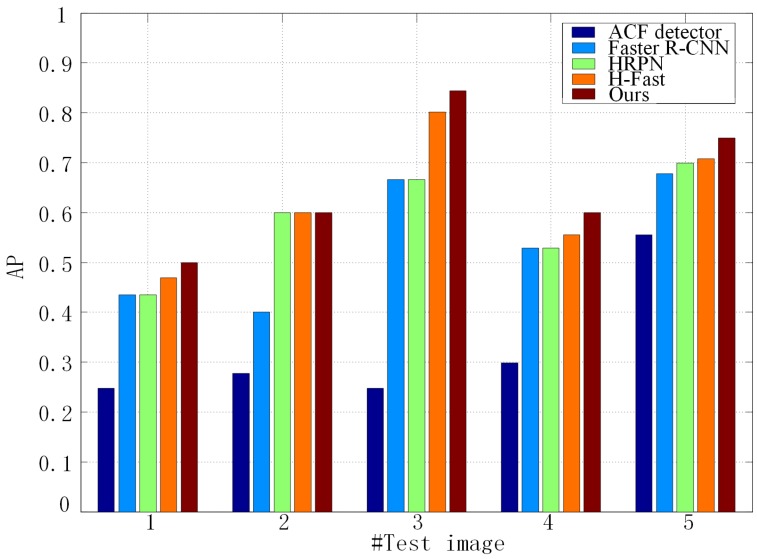
Performance comparisons of different methods in terms of AP values for the collected vehicle dataset.

To show the robustness of our method, we also evaluate it on the collected vehicle data set. We directly use the model trained on the Munich dataset and test the image without training on this new dataset. Table 5 and Figure 11 show the results of different methods tested on the collected vehicle data set. In Table 5, the best performances are highlighted in bold. Our proposed method still achieves the best performance in terms of recall rate, precision and F1-Score. These results also indicate that CNN based methods have better migration capacity compared with the traditional method. Figure 11 shows detection AP of different methods for five pictures. The histogram shows that our method still has higher AP than other methods for the new dataset.

**Figure 12:**
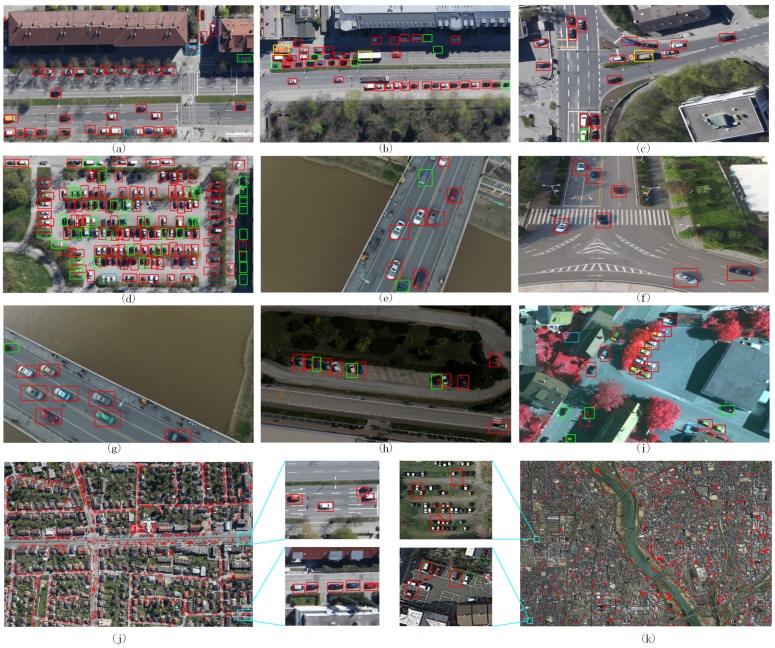
Detection results from test images. Red boxes denote correct localization of car, yellow boxes denote correct localization of truck, green boxes and blue boxes denote missing detection and incorrect detection, respectively. (**a**–**d**) are results for the Munich test aerial image blocks; (**e**–**h**) are results on unmanned aerial vehicle (UAV) images; (**i**) is results for pansharpened color infrared (CIR) image; (**j**) is results for the original large-scale Munich test image; (**k**) is results for a large satellite image of Tokyo.

Figure 12a–d show several vehicle detection results of test image blocks on the Munich dataset with our proposed method, in which the red box denotes correct localization of car, the yellow box denotes correct localization of truck, and the green box and blue box denote missing detection and wrong detection, respectively. Figure 12j shows detection results for original large-scale test images (5616×3744 pixels). As shown in Figure 12, despite the vehicles being located in the shade or near the image block boundaries (only part of the vehicle is shown in the image), the proposed approach has successfully detected most of the vehicles. Figure 12e–i show several vehicle detection results with the proposed approach on the collected vehicle dataset. Figure 12k shows results for a very large satellite image (18239×12837 pixels) of Tokyo download from Google Earth, Mountain View, CA, USA. It can be observed that our method can detect most of the vehicles successfully from the collected vehicle dataset but performs poorly for such large satellite images. These results demonstrate that our method has good migration capacity for some images, but not all of the images. Moreover, our method cannot handle highly occluded vehicles (see Figure 12d,h), and the vehicles in thick shadows (see Figure 12a,d). This may be caused by the overlap of the vehicles’ local peaks in the coarse hyper feature map. In a future study, we will consider enhancing the resolution of the hyper feature map and improve the migration capacity of our method.

## 5. Conclusions

In this paper, we developed a vehicle detector with two parts: an HRPN to generate vehicle-like regions, which combines hierarchical feature maps for small object detection and a cascade of boosted classifiers, which improves the classification accuracy by hard negative example mining. Extensive experiments on two datasets show that our method can detect small-sized vehicles more accurately and reject most of the vehicle-like background objects. It also has good robustness for images captured from UAV or CIR. For future work, the performance could be further improved by enhancing the resolution of the hyper feature map using deconvolution layers to improve the detection performance further.

## Figures and Tables

**Table 1:** The mean average precision (mAP) under different scales and aspect ratios of anchors.

Settings	Anchor Scale	Aspect Ratios	mAP
	$30^{2}$	1:1	0.7624
**1 scale, 1 ratio**	$40^{2}$	1:1	0.7625
	$50^{2}$	1:1	0.7623
	$30^{2}$	1:1, 1:2, 2:1	0.7950
**1 scale, 3 ratio**	$40^{2}$	1:1, 1:2, 2:1	0.7951
	$50^{2}$	1:1, 1:2, 2:1	0.7950
**3 scale, 1 ratio**	$30^{2}$, $40^{2}$, $50^{2}$	1:1	0.7624
**3 scale, 3 ratio**	$30^{2}$, $40^{2}$, $50^{2}$	1:1, 1:2, 2:1	0.7954

**Table 2:** Number of each type of vehicle.

Type	Number	Type	Number
car	3191	car _ trail (car with trailer)	1
van	227	van _ trail (van with trailer)	3
truck	57	truck _ trail (truck with trailer)	7
cam (long truck)	8	bus	8

**Table 3:** Recall and mean average precision (mAP) with different layers.

Layers	Recall	mAP
3	68.60%	66.46%
4	69.31%	68.29%
5	72.40%	72.92%
34	73.29%	73.11%
45	73.58%	73.33%
345	78.30%	79.54%

**Table 4:** Performance comparison between different methods.

Method	Ground Truth	True Positive	False Positive	Recall Rate	Precision Rate	F1-Score	Time/per Image
ACF detector	5892	3078	4062	52.24%	43.31%	0.47	4.37
Faster R-CNN	5892	4050	503	68.74%	88.95%	0.78	3.84
H-Fast	5892	4363	696	74.00%	86.2%	0.80	3.65
Ours	5892	4615	560	78.3%	89.2%	0.83	3.93

**Table 5:** Results of different methods for the collected vehicle images

Method	Ground Truth	True Positive	False Positive	Recall Rate	Precision Rate	F1-Score
ACF detector	882	382	652	43.31%	36.94%	0.3987
Faster R-CNN	882	395	149	44.78%	72.61%	0.5540
HRPN	882	400	145	45.35%	73.39%	0.5605
H-Fast	882	411	169	46.60%	70.86%	0.5622
Ours	882	439	146	49.77%	75.04%	0.5985
